# Correction to ‘Late Pleistocene shell midden microstratigraphy indicates a complex history of human–environment interactions in the uplands of North Vietnam’

**DOI:** 10.1098/rstb.2022.0092

**Published:** 2022-06-20

**Authors:** Conor McAdams, Mike W. Morley, Xiao Fu, Alexander V. Kandyba, Anatoly P. Derevianko, Dong Truong Nguyen, Nguyen Gia Doi, Richard G. Roberts

*Phil. Trans. R. Soc. B* **377**, 20200493. (Published 7 March 2022) (doi:10.1098/rstb.2020.0493)

The originally published version of this paper used the term ‘North Vietnam’ to refer to northern Vietnam and not the former Democratic Republic of Vietnam. For clarity, this term has been changed to ‘northern Vietnam’.

